# A four-factor model of executive function: Predicting physical and academic outcomes from cognitive assessments in adolescents

**DOI:** 10.1016/j.dcn.2024.101471

**Published:** 2024-10-30

**Authors:** Alejandro D. Meruelo, Tommy Gunawan, Michael L. Thomas, Vijay A. Ramchandani

**Affiliations:** a University of California, San Diego, 9500 Gilman Dr, La Jolla, CA 92093, USA; b Human Psychopharmacology Laboratory and Office of Clinical Director, Division of Intramural Clinical and Biological Research, National Institute on Alcohol Abuse and Alcoholism, Bethesda, MD 20817 USA; c Colorado State University, 1876 Campus Delivery, Fort Collins, CO 80523-1876, USA; d Human Psychopharmacology Laboratory, Division of Intramural Clinical and Biological Research, National Institute on Alcohol Abuse and Alcoholism, Bethesda, MD 20817, USA

**Keywords:** Impulsivity, Cognition, Confirmatory factor analysis, Body-mass index, Physical activity, Academic performance

## Abstract

Impulsivity and cognitive function are essential for understanding behavioral regulation, particularly in relation to health-risk behaviors like substance use, physical activity, and academic performance. This study examined the factor structure underlying executive function in adolescents using the UPPS-P Impulsive Behavior Scale and NIH Toolbox Cognition Battery. We explored how parental monitoring moderates, and peer network health and perceived stress mediate, relationships between cognitive function and outcomes such as BMI, physical activity, and academic performance. Exploratory factor analysis (EFA) on 2228 observations identified a four-factor model (BIC = −97.92, RMSEA = 0.040, TLI = 0.936), validated by confirmatory factor analysis (CFA) (CFI = 0.961, RMSEA = 0.055). Structural equation modeling (SEM) on 5902 observations showed that parental monitoring moderated Factor 1 (adaptive impulsivity) in relation to physical activity and academic performance, while peer network health mediated Factor 2 (emotional impulsivity) effects on BMI and physical activity. This model underscores the influence of peer relationships, parental involvement, and stress on cognitive, health, and academic outcomes, suggesting that interventions enhancing peer support, reducing stress, and promoting healthy behaviors may improve adolescent well-being.

## Highlights

• A four-factor model of impulsivity and cognition was identified and validated.
• Parental monitoring moderated the impact of impulsivity on physical activity & grades.
• Peer network health mediated emotional impulsivity's effect on BMI & activity.
• Factor 4 (attention/executive function) was linked to BMI & activity.

## Graphical Abstract

**Figure:**
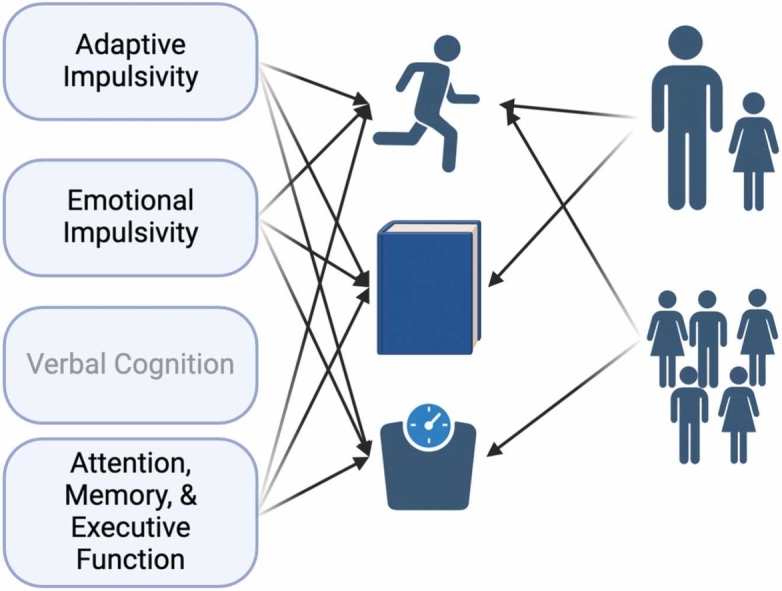

## 1 Introduction

Executive function is a critical cognitive domain that underpins the regulation of behavior, decision-making, and goal-directed activity. Understanding the factors that contribute to the development of executive function, especially during adolescence, is crucial due to its significant role in shaping long-term health behaviors(Allan et al., 2016). The Addictions Neuroclinical Assessment (ANA)(Kwako et al., 2019, Kwako et al., 2016) model offers a robust framework for examining executive function, incorporating measures such as NIH Toolbox cognitive assessments(Holdnack et al., 2017; Shields et al., 2020; Weintraub et al., 2014) and impulsivity-related scales like the UPPS-P impulsive behavior scale(Behrouzian et al., 2022; Cyders et al., 2014; Hasegawa et al., 2020; Samiefard et al., 2023; Xue et al., 2017). These diverse components capture essential aspects of cognitive control and impulse regulation.

Previous research has highlighted the importance of NIH Toolbox cognitive measures providing comprehensive insights into various dimensions of cognitive ability(Allan et al., 2016; Holdnack et al., 2017). The NIH Toolbox cognition battery includes several standardized tests that measure critical cognitive abilities such as executive function, attention, memory, language, and processing speed. The assessment features engaging and interactive tasks, including the Flanker Task for attention and inhibition, the Picture Vocabulary Test for verbal comprehension(Weintraub et al., 2013), and the Pattern Comparison Task for processing speed.

Impulsivity, as measured by the UPPS-P scale (Urgency, Premeditation, Perseverance, Sensation Seeking, and Positive Urgency), adds another layer of complexity to our understanding of executive function. Impulsivity is a multifaceted construct that significantly influences various behavioral outcomes in adolescents(Aoun et al., 2019; Arens et al., 2012; Babinski et al., 1999; Bakhshani, 2014; Hinojosa-Aguayo and González, 2020), including academic performance and physical activity(Alavi et al., 2019; Marriott et al., 2019). Recent operationalization of impulsivity (e.g., the UPPS-P scale)(Aoun et al., 2019; Bakhshani, 2014; Hinojosa-Aguayo and González, 2020), suggest that negative urgency, lack of planning, and positive urgency may differentially affect how individuals navigate their social environments and respond to stressors. Understanding these dimensions is crucial, as impulsivity has been linked to adverse outcomes, particularly in vulnerable populations(Babinski et al., 1999; Bakhshani, 2014).

In this study, we conduct an exploratory factor analysis (EFA)(Gaskin and Happell, 2014) and subsequent confirmatory factor analysis (CFA) to elucidate and validate the latent factors underlying executive function by integrating NIH Toolbox cognitive measures and UPPS-P impulsivity scores. Building on this, we used data derived from year 1 to year 5 of the Adolescent Brain Cognitive Development (ABCD) study(“About the Study,” 2023; “Baseline Data Demographics 2.0,” n.d.; “NIH’s Adolescent Brain Cognitive Development (ABCD) Study,” 2018; Garavan et al., 2018; Volkow et al., 2018) to investigate the roles of these factors on adolescent outcomes such as academic performance and engagement in physical activity and body-mass index through different mediating and moderating factors reflecting individual traits and social context. Peer relations(Aljehani et al., 2022; Alswat et al., 2017; Darling-Hammond and Cook-Harvey, 2018; Franz and Feresu, 2013; Kwong and Davis, 2015; Zax and Rees, 2002) may have mediating roles in the prediction of these outcomes based on these latent factors. Parental involvement has been consistently associated with the promotion of positive behaviors in adolescence(Pelham et al., 2024), and its role in moderating the relationship between early cognitive abilities and physical (e.g., body-mass index, physical activity) and academic outcomes remains understudied.

Through this approach, we aim to identify cognitive and psychosocial factors that influence executive function development and long-term health behaviors, providing insights into potential intervention points to enhance adolescent well-being.

## 2 Materials and methods

### 2.1 Participants

The sample for this study was derived from the Adolescent Brain Cognitive Development (ABCD) study (“About the Study,” 2023; “Baseline Data Demographics 2.0,” n.d.; “NIH’s Adolescent Brain Cognitive Development (ABCD) Study,” 2018; Garavan et al., 2018; Volkow et al., 2018), a nationwide longitudinal cohort that recruited adolescents aged 9–10, defined as the baseline year, from 21 research sites across the United States and is tracking their development over 10 years. The ABCD study aims to better understand the factors influencing brain, cognitive, and behavioral development throughout adolescence, with a focus on how various environmental, genetic, and social factors impact health outcomes. For the current analysis, data were obtained across a range of years described below of the ABCD dataset. The initial, unsplit sample included 4455 observations corresponding to complete data for both cognitive and impulsivity measures, and additional data on variables for our mediation-moderation analyses (Table 1, Table 2**; see Supplemental Figure 1 for schematic of data collection by year**). The total sample was split into two subsamples: one for the exploratory factor analysis (EFA; n = 2227) and another for the confirmatory factor analysis (CFA; n = 2228). For the subsequent mediation and moderation analyses, the sample consisted of 5902 observations (mean age at year 2: 12.90 years, SD: 0.65; 48 % female) with included measures of impulsivity, cognitive functioning, body-mass index (BMI), physical activity, academic performance, Perceived Stress, Parental Monitoring, and peer relationships. Institutional review board (IRB) approval was obtained at each research site, and informed consent was provided by parents or legal guardians, while assent was obtained from the adolescents **(**Table 2).

**Table 1:** Key Variables for Executive Function Factor Analyses (N=4455).

**Variable**	**Mean**	**SD**
UPPS Lack of Perseverance	6.87	2.24
UPPS Lack of Planning	7.73	2.23
UPPS Positive Urgency	7.27	2.72
UPPS Negative Urgency	7.72	2.36
NIH Tbx Picture Vocabulary	9.61	2.69
NIH Tbx Flanker	96.49	13.64
NIH Tbx List	101.93	14.32
NIH Tbx Pattern	108.52	17
NIH Tbx Reading	103.28	18.32
NIH Tbx Card Sort	94.66	21.51
Delayed Discounting K-value	0.20	1.35

Tbx = Toolbox

**Table 2:** Key Variables for Mediation and Moderation Model (N= 5902).

**Variable**	**Mean**	**SD**	**Min**	**Max**	**Percentage**
Sex (% Female)					47.98
NIH Tbx Flanker (baseline; age-corrected)	96.53	13.66	62	151	
NIH Tbx List (baseline; age-corrected)	101.98	14.33	59	181	
NIH Tbx Picture Vocabulary Score (baseline; age-corrected)	108.48	16.98	1	193	
NIH Tbx Reading (baseline; age-corrected)	103.31	18.24	68	189	
NIH Tbx Pattern (baseline; age-corrected)	94.75	21.49	20	177	
NIH Tbx Card Sort (baseline; age-corrected)	97.89	15.64	68	181	
Parent Monitoring Scale (Year 1)	4.80	0.47	1	5	
UPPS Lack of Perseverance (Year 2)	6.84	2.24	4	16	
UPPS Lack of Planning (Year 2)	7.71	2.22	4	16	
UPPS Positive Urgency (Year 2)	7.25	2.71	4	16	
UPPS Negative Urgency (Year 2)	7.70	2.35	4	16	
UPPS Sensation Seeking (Year 2)	9.62	2.69	4	16	
Age in Years (Year 2)	12.90	0.65	11.42	14.42	
Body-Mass Index (Year 2)	20.44	4.86	5.75	47.43	
Peer Network Health (Year 2)	11.87	7.84	0	27	
Perceived Stress (Year 3)	5.96	54.78	0	777	
Grades in Past Year (Year 3)	3.40	2.04	1	12	
Physical Activity Score (Year 5)	2.41	1.57	0	5	

Tbx = Toolbox

### 2.2 Demographics

The ABCD study gathered comprehensive demographic information, including participants' age, sex, ethnicity, household income, and family identification. Household income data were obtained from the ABCD Longitudinal Parent Demographics Survey(“Baseline Data Demographics 2.0,” n.d.). Income levels were categorized using a 10-point scale, ranging from 1 (less than $5000) to 10 ($200,000 and greater). The scale provided detailed income brackets, such as $5,000-$11,999 (2), $12,000-$15,999 (3), and so on, up to $100,000-$199,999 (9). Household income was normalized by number of household members. To facilitate family-based analyses, each participant was assigned a unique family ID, with siblings or related participants sharing the same identifier.

### 2.3 Executive function and impulsivity

Executive function and impulsivity were assessed using a combination of NIH Toolbox cognitive tests and the UPPS-P Impulsive Behavior Scale. The following measures were included:

#### 2.3.1 NIH toolbox cognition battery

This is a comprehensive assessment tool(Holdnack et al., 2017; Weintraub et al., 2014, Weintraub et al., 2013) designed to evaluate cognitive function across various domains in individuals aged 3–85 years. This battery encompasses several standardized tests that measure key cognitive abilities, including executive function, attention, memory, language, and processing speed. The assessment employs a variety of engaging and interactive tasks, such as the Flanker Task for assessing attention and inhibition, the Picture Vocabulary Test for evaluating verbal comprehension, and the Pattern Comparison Task to measure processing speed. Each test is designed to be brief and adaptable to accommodate participants of different ages and cognitive levels, making it suitable for diverse research contexts. The total duration of time to complete the battery by a participant is between 30 and 40 minutes. In this study, the NIH Toolbox Cognition Battery was administered to assess participants' cognitive functioning, facilitating the exploration of its associations with impulsivity and behavioral outcomes. Baseline assessments were utilized for our analyses.

#### 2.3.2 UPPS-P impulsive behavior scale

This is a widely used self-report questionnaire(Behrouzian et al., 2022; Cyders et al., 2014; Hasegawa et al., 2020; Samiefard et al., 2023; Xue et al., 2017) designed to assess various dimensions of impulsivity, including five key factors: Lack of Perseverance, Lack of Planning, Positive Urgency, Negative Urgency, and Sensation Seeking. Each factor reflects distinct impulsive behaviors and tendencies, with Lack of Perseverance indicating difficulty in sustaining attention and effort; Lack of Planning representing a tendency to act without forethought; Positive Urgency capturing impulsive reactions to positive emotions; Negative Urgency reflecting impulsive responses to negative emotions; and Sensation Seeking representing a preference for excitement and novel experiences. The scale consists of a series of items rated on a Likert-type scale, where higher scores signify greater impulsivity in each domain. In this study, the UPPS-P was utilized to explore its relationship with cognitive and behavioral measures among participants, providing valuable insights into the role of impulsivity and sensation seeking in various contexts.

#### 2.3.3 Academic performance assessment

The evaluation of academic performance was based on students' grades from the preceding academic year (Youth School Attendance and Grades data(Li et al., 2024)). The grading system employed a self-reporting method, with participants providing their own grades. These were then converted to a numerical scale ranging from 1 to 12, where lower numbers corresponded to higher academic achievement. The scale was structured as follows: 1 = A+, 2 = A, 3 = A-, 4 = B+, 5 = B, …, 12 = F. Our study utilized participant data from Year 3.

#### 2.3.4 Body mass index (BMI)

BMI was calculated from the provided heights and weights of participants at Year 2 using the formula: BMI = 703 * weight in pounds/height * height in inches squared.

#### 2.3.5 Physical activity via youth risk behavior survey

Physical activity at Year 5 was measured using self-reported responses from the Youth Risk Behavior Survey (YRBS)(“Methodology of the Youth Risk Behavior Surveillance System,” 2004), which assessed the frequency and intensity of physical activity over the previous week. Participants were asked to report the number of days they engaged in moderate-to-vigorous physical activity for at least 60 minutes.

#### 2.3.6 Parental monitoring scale

Parental Monitoring at Year 1 was assessed using the Parental Monitoring Scale(Stattin and Kerr, 2000), which included items measuring the extent to which parents or guardians kept track of the adolescent’s whereabouts, activities, and friendships. Higher scores reflected greater levels of parental supervision and involvement.

#### 2.3.7 Perceived stress

The Perceived Stress Scale(Cohen et al., 1983) at Year 3 was used to assess participants' levels of stress over the past month. This scale included the item "In the last month, how often have you felt nervous and 'stressed'?" measured on a Likert scale with response options ranging from 0 (Never) to 4 (Very Often). Responses were classified into five categories from no stress (0) to high stress (4).

#### 2.3.8 Peer network health

The Peer Network Health(Mason et al., 2004a) at Year 2 was utilized to assess the protective aspects of participants' peer networks. This scale calculates a composite score by summing responses from about each peer's substance use, influence on behavior, and types of activities. Participants reported on negative or risky behaviors, including whether they knew if each nominated peer used substances, if the peer was a daily user, and whether they were influenced by each peer to use or not use substances. They also indicated involvement in illegal, violent, or dangerous activities. Additionally, participants shared details about positive or protective activities with their peers, such as receiving help with schoolwork or transportation and providing support through discussions of problems. This information contributes to a total score for each peer based on a weighted scoring system that ranges from −14–14. Higher scores indicate greater Peer Network Health, while lower scores suggest increased behavioral risk. This scale demonstrates favorable internal reliability with a Cronbach’s alpha of 0.84 and shows significant correlations with self-reported substance use measures, including alcohol and marijuana use(Mason et al., 2004b).

#### 2.3.9 Delayed discounting task

The delayed discounting task(McKerchar et al., 2009; Odum, 2011) is a widely used measure of impulsivity that assesses an individual's preference for smaller immediate rewards versus larger delayed rewards. In the ABCD study, participants completed a computerized delayed discounting task at Year 1 in which they were asked to make a series of choices between an immediate monetary reward (e.g., $10 now) and a larger delayed reward (e.g., $50 in 30 days). The delayed discounting paradigm evaluates the degree to which participants discount the value of the delayed reward, capturing impulsive decision-making tendencies.

### 2.4 Calculation of discounting rates (k-values)

To quantify the rate at which participants discounted future rewards, we applied the Mazur (1987) method(Mazur, 1988; McKerchar et al., 2009; Odum, 2011), which models the relationship between reward size and delay. The Mazur method is based on a hyperbolic discounting function, where the subjective value (V) of a delayed reward (A) decreases as a function of the delay (D) and the discount rate (k), represented by the equation: V = A/(1+kD)

The k-value, a key parameter of this model, reflects the individual participant’s rate of discounting. Higher k-values indicate a greater preference for immediate rewards (i.e., higher impulsivity), while lower k-values suggest a greater tolerance for delayed rewards. For each participant, k-values were estimated using maximum likelihood estimation, allowing for individualized measures of impulsivity derived from their choices across varying reward-delay pairs.

These k-values were then included in our exploratory factor analysis (EFA) to investigate their relationship with other cognitive and impulsivity measures, including the UPPS-P subscales and NIH Toolbox assessments.

### 2.5 Exploratory factor analysis (EFA)

To explore the underlying factor structure of executive function, we conducted EFA on the first subsample (n = 2227). Factors were extracted using the minimum residual (minres) method with varimax rotation. Models ranging from two to four factors were compared using Bayesian Information Criterion (BIC), Root Mean Square Error of Approximation (RMSEA), and Tucker-Lewis Index (TLI); cognitive and impulsivity measures were approximately normally distributed. The number of factors to retain was determined using parallel analysis and scree plots. The best-fitting solution was selected based on goodness-of-fit indices. An ideal fit is typically defined as having an RMSEA ≤ 0.06, CFI and TLI ≥ 0.95(Cangur and Ercan, 2015) and a good fit as having CFI and TLI ≥ 0.90(Schreiber et al., 2006), independent of sample size. Factor loadings greater than 0.3 were considered important, and items with high cross-loadings or low communalities were carefully evaluated for model retention.

### 2.6 Confirmatory factor analysis (CFA)

CFA was conducted on the second subsample (n = 2228) to validate the factor structure identified by the EFA. The CFA evaluated model fit using indices such as the Comparative Fit Index (CFI) and RMSEA.

### 2.7 Mediation and moderation structural equation model analyses

To investigate mediation and moderation of a variety of psychological and environmental factors of the relationship between executive function and physical activity, we utilized the R package lavaan(Rosseel, 2012).

SEM was performed on the full sample (n = 5902) to investigate the pathways through which cognitive functioning and impulsivity impact health and academic outcomes. The model included Parental Monitoring as a moderator and Perceived Stress and peer relationships as mediators. The four-factor structure identified in the EFA and CFA was initially incorporated into the SEM model. However, Factor 3 (verbal cognition) was removed from the SEM analysis due to a lack of model convergence. Cross-loading on lack of perseverance was utilized between factors 1 and 2 to improve model fit. The final SEM model examined the effects of Factors 1 (adaptive impulsivity), 2 (emotional impulsivity), and 4 (attention and memory) on BMI, physical activity, and academic performance. The model was adjusted for relevant covariates, including age, sex, normalized household income, and family identity (e.g., two participants belonging to same family).

Confidence intervals were computed using the percentile method, and statistical significance was evaluated at the 0.05 level.

### 2.8 Statistical Analysis

All analyses were performed using R version 4.2.2. Descriptive statistics were calculated to summarize sample demographics and study variables. Missing data were addressed using listwise deletion. Results were considered statistically significant at p < 0.05.

## 3 Results

### 3.1 Exploratory factor analysis of executive function (Table 3, Table 4, Table 5, Table 6)

**Fig. 1:**
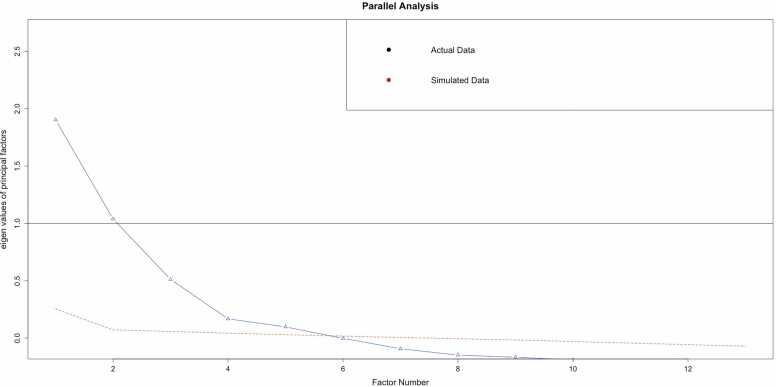
**:** Parallel Analysis Scree Plot. The scree plot favored a four-factor solution based on the elbow in the curve.

To identify the latent structure of executive function, we conducted an exploratory factor analysis (EFA) on the half of the initial dataset, using measures from the NIH Toolbox cognitive assessments, UPPS-P impulsivity scores, and delayed discounting k-values. The EFA was performed on a sample of 2227 observations. The analysis explored models with 2–4 factors (parallel analysis scree plot shown in Fig. 1**; Supplemental Tables 1 and 2 showing 2- and 3- Factor analyses**), with the fit statistics favoring a four-factor solution over the two-factor and three-factor models.

**Table 3:** Overview of Models with Goodness-of-Fit Indices.

**Number of Factors**	**BIC**	**Degrees of Freedom**	**p-value**	**TLI**	**RMSEA**
2	787.23	53	< 0.001	0.623	0.098
3	268.8	42	< 0.001	0.771	0.077
4	−97.92	32	< 0.001	0.936	0.040

**Table 4:** Factor Loadings for the 4-Factor Solution.

**Measure**	**Factor 1**	**Factor 2**	**Factor 3**	**Factor 4**
UPPS Lack of Perseverance	−0.1	0.99	0.06	0
UPPS Positive Urgency	−0.17	0.15	0.77	−0.05
UPPS Sensation Seeking	0.13	−0.06	0.37	0.02
UPPS Negative Urgency	−0.06	0.21	0.6	−0.02
UPPS Lack of Planning	−0.01	0.43	0.3	0.02
NIH Tbx Picture Vocabulary (Age Corrected)	0.73	−0.01	0	0.05
NIH Tbx Flanker (Age Corrected)	0.18	−0.01	0.04	0.58
NIH Tbx List (Age Corrected)	0.52	−0.06	0	0.2
NIH Tbx Card Sort (Age Corrected)	0.24	−0.07	0.03	0.67
NIH Tbx Pattern (Age Corrected)	0.14	−0.05	0.01	0.56
NIH Tbx Picture (Age Corrected)	0.32	−0.07	−0.03	0.22
NIH Tbx Reading (Age Corrected)	0.63	0	0.01	0.1
Delayed Discounting K-value	0	0.01	−0.01	0.03

NIH Tbx - NIH Toolbox

**Table 5:** Confirmatory Factor Analysis Results.

**Statistic**	**Value**
Chi-square Test Statistic	262.623
Degrees of Freedom (df)	38
P-value (Chi-square)	0.000
Comparative Fit Index (CFI)	0.945
Tucker-Lewis Index (TLI)	0.920
Akaike Information Criterion (AIC)	160,615.429
Bayesian Information Criterion (BIC)	160,775.277
Sample-size adjusted BIC (SABIC)	160,686.316
Root Mean Square Error of Approximation (RMSEA)	0.052
90 % CI (RMSEA lower)	0.046
90 % CI (RMSEA upper)	0.057
Standardized Root Mean Square Residual (SRMR)	0.045

**Table 6:** Confirmatory Factor Analysis Latent Variable Loadings.

**Latent Variables**	**Observed Variables**	**Estimate**	**SE**	**z-value**	**P-value**	**Std.lv**	**Std.all**
Factor 1 (Adaptive Impulsivity)	UPPS Lack of Perseverance	1.000	-	-	-	1.322	0.571
	UPPS Lack of Planning	1.397	0.119	11.771	0.000	1.847	0.829
Factor 2 (Emotional Impulsivity)	UPPS Positive Urgency	1.000	-	-	-	2.048	0.762
	UPPS Negative Urgency	0.762	0.047	16.151	0.000	1.561	0.668
	UPPS Sensation Seeking	0.337	0.034	9.918	0.000	0.690	0.265
Factor 3 (Verbal Cognition)	NIH Tbx Picture Vocabulary (Age Corrected)	1.000	-	-	-	11.581	0.683
	NIH Tbx List (Age Corrected)	0.662	0.036	18.202	0.000	7.666	0.538
	NIH Tbx Reading (Age Corrected)	1.137	0.059	19.238	0.000	13.168	0.699
Factor 4 (Attention, Memory, and Executive Function)	NIH Tbx Flanker (Age Corrected)	1.000	-	-	-	8.356	0.617
	NIH Tbx Card Sort (Age Corrected)	1.323	0.073	18.037	0.000	11.059	0.720
	NIH Tbx Pattern (Age Corrected)	1.434	0.081	17.615	0.000	11.982	0.546

NIH Tbx - NIH Toolbox

SE = Standard Error

Std.lv standardizes the latent factors in the model, while keeping the observed variables in their original scale. Std.all, standardizes both the latent and observed variables.

#### 3.1.1 Factor structure for the four-factor model (Table 4)

The four-factor solution demonstrated an excellent fit, with 32 degrees of freedom for the model and an objective function value of 0.07. The root mean square of the residuals (RMSR) was 0.02, and the df-corrected RMSR was 0.03, indicating a very good fit. The RMSEA index was 0.04, with a 90 % confidence interval ranging from 0.034 to 0.047, further suggesting a strong model fit. The Tucker-Lewis Index (TLI) of factoring reliability was 0.936, and the Bayesian Information Criterion (BIC) was −97.92. Additionally, the fit based upon off-diagonal values was 0.99.

Factor 1 captured variance primarily in verbal cognition from the NIH Toolbox Cognition Battery, such as Picture Vocabulary and Reading, with loadings ranging from 0.52 to 0.73. Factor 2 was associated with adaptive impulsivity measures from the UPPS-P Impulsive Behavior Scale, including Lack of Perseverance and Lack of Planning, with loadings of 0.99 and 0.43, respectively. Factor 3 was linked to emotional impulsivity measures, such as Positive Urgency and Negative Urgency, with loadings of 0.77 and 0.60, respectively. Factor 4 captured variance in memory/attention/executive function measures like Flanker and Card Sort, with loadings of 0.58 and 0.67. These four factors accounted for 39 % of the total variance, with Factor 1 explaining 11 %, Factor 2 contributing 10 %, Factor 3 explaining 9 %, and Factor 4 explaining another 9 %.

These results indicate a meaningful distinction between the verbal cognition, adaptive impulsivity, emotional impulsivity, and attention/memory/executive function domains captured by the four factors.

#### 3.1.2 Comparison and interpretation

Across all three models, a clear structure emerged that distinguished impulsivity-related traits from cognitive domains. The introduction of additional factors improved the differentiation between types of impulsivity, reflecting adaptability and emotionality, and specific cognitive abilities, such as verbal comprehension, attention, and executive function. Based on the scree plot and parallel analysis, the EFA favored a four-factor solution with good fit statistics. The four factors highlighted the multidimensionality of two major types of impulsivity, verbal comprehension/language, and attention/memory/ executive function in our sample. The factor correlations were modest but significant, suggesting some degree of overlap between these domains, although they remained largely independent constructs.

These findings suggest that the adaptive and emotional impulsivity, verbal comprehension/language, and attention/ memory/executive function are best conceptualized as distinct but related factors, with separate dimensions underlying different aspects of behavior and cognition. The four-factor solution, in particular, allowed for a more granular understanding of these constructs, supporting its use in subsequent analyses.

### 3.2 Confirmatory factor analysis

The CFA on a sample of 2228 observations, using Maximum Likelihood (ML) estimation and the NLMINB optimization method, resulted in a good model fit (CFI and TLI ≥ 0.90, RMSEA within an acceptable range with a lower CI of 0.046 and upper CI of 0.057) and shown in Table 5, Table 6:

All factor loadings were statistically significant (p < 0.001), indicating a strong relationship between the latent factors and their respective observed variables.

The CFA results confirmed the factor structure identified in the EFA, with good fit indices (CFI = 0.945, TLI = 0.920, RMSEA = 0.052). The factors represented adaptive impulsivity, emotional impulsivity, verbal cognition, and memory/attention/executive functioning, with significant factor loadings and relationships among factors.

Significant covariances were observed between several factors (Table 7), indicating relationships between Factor 1 (Adaptive Impulsivity) and Factor 2 (Emotional Impulsivity), and negative correlations between Factor 2 (Emotional Impulsivity) and Factor 3 (Verbal Cognition) and Factor 4 (Attention, Memory, and Executive Function).

**Table 7:** Confirmatory Factor Model Covariances:.

**Latent Variable Pairs**	**Estimate**	**SE**	**z-value**	**P-value**	**Std.lv**	**Std.all**
Factor 1 ∼∼ Factor 2	1.287	0.127	10.113	0.000	0.475	0.475
Factor 2 ∼∼ Factor 3	−3.566	0.741	−4.810	0.000	−0.150	−0.150
Factor 2 ∼∼ Factor 4	−2.028	0.537	−3.778	0.000	−0.119	−0.119
Factor 3 ∼∼ Factor 4	42.774	3.674	11.641	0.000	0.442	0.442

SE = Standard Error

Std.lv standardizes the latent factors in the model, while keeping the observed variables in their original scale. Std.all, standardizes both the latent and observed variables.

### 3.3 Mediation and moderation structural equation model for factors 1, 2, and 4 (Table 8 and Fig. 2)

The model demonstrated a good fit, with a Comparative Fit Index (CFI) of 0.961 and a Tucker-Lewis Index (TLI) of 0.933, indicating a strong model fit relative to the baseline model. The Root Mean Square Error of Approximation (RMSEA) was 0.055, with a 90 % confidence interval ranging from 0.052 to 0.058, suggesting that the model’s residuals were small, and the fit was adequate. The Standardized Root Mean Square Residual (SRMR) of 0.043 further supports the model’s satisfactory fit. The model estimated a total of 64 parameters based on 5902 observations, with a chi-square value of 1482.532 and 72 degrees of freedom, reflecting significant differences from the null model, which is expected given the complexity of the model.

**Table 8:** Summary Statistics for Mediation-Moderation Structural Equation Model.

**Latent Variable**	**Indicator**	**Estimate**	**SE**	**z-value**	**P**	**Std.lv**	**Std.all**
Factor 1	UPPS Lack of Perseverance	1				1	1.009
Factor 1	UPPS Positive Urgency	1.611	0.061	26.484	0	1.611	0.592
Factor 2	UPPS Negative Urgency	1				1.007	1.01
Factor 2	UPPS Lack of Perseverance	−0.571	0.043	−13.169	0	−0.575	−0.581
Factor 4	NIH Tbx Flanker (Age Corrected)	1				0.586	0.586
Factor 4	NIH Tbx Card Sort (Age Corrected)	1.276	0.049	25.997	0	0.748	0.749
Factor 4	NIH Tbx Pattern (Age Corrected)	0.943	0.033	28.182	0	0.553	0.554

SE = Standard Error

Std.lv standardizes the latent factors in the model, while keeping the observed variables in their original scale. Std.all, standardizes both the latent and observed variables.

Factor loadings for the latent variables were mostly significant and well-defined. For Factor 1, the loadings were strong, with UPPS Lack of Perseverance as the primary indicator (1.000) and UPPS Positive Urgency loading at 1.611 (p < 0.001). Factor 2 was defined by UPPS Negative Urgency (1.000) and UPPS Lack of Perseverance (-0.571, p < 0.001), showing significant cross-loadings between factors. For Factor 4, the cognitive indicators from the NIH Toolbox, including NIH Toolbox Flanker, NIH Toolbox Card Sort, and NIH Toolbox Pattern, demonstrated strong factor loadings ranging from 0.586 to 0.748 (all p < 0.001).

**Fig. 2:**
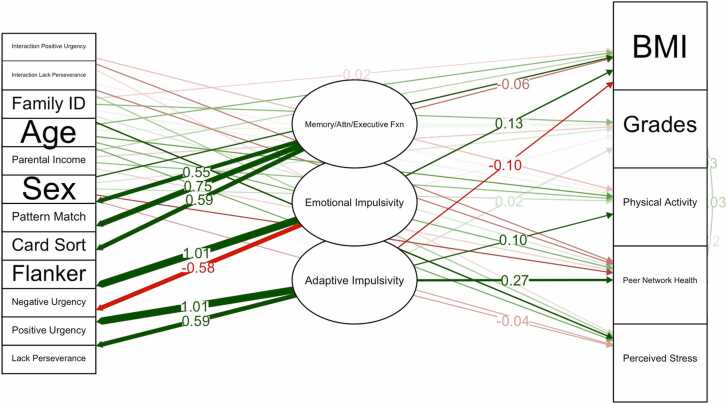
**:** Path Diagram for Structural Equation Mediation-Moderation Model. A path diagram of the mediation-moderation structural equation model highlighting the role of impulsivity traits and cognitive functioning in shaping physical activity, academic performance, and BMI.

In the regressions, Factor 1 showed a significant positive relationship with Past Year Grades (Estimate = 0.267, p < 0.001) and BMI (Estimate = 0.097, p < 0.001). It also showed a significant negative effect on Physical Activity (Estimate = −0.036, p = 0.010). Interaction terms involving Factor 1 influenced outcomes, such as the interaction between impulsivity traits and physical activity (Estimate = 0.033, p = 0.006). Perceived Stress was not significantly associated with physical activity but showed a significant positive effect on Peer Network Health (Estimate = 0.004, p = 0.015) and a positive association with Past Year Grades (Estimate = 0.004, p = 0.010).

Overall, the model results suggest that the cross-loadings between the impulsivity-related and cognitive factors, as well as the interactions involving latent factors, provide meaningful contributions to predicting behavioral, academic, and health outcomes. These findings underscore the importance of both impulsivity traits and cognitive functioning in shaping physical activity, academic performance, and BMI (Fig. 2).

## 4 Discussion

This study aimed to explore the underlying factor structure of executive function and impulsivity, as well as to examine how cognitive abilities measured early in adolescence mediate and moderate physical activity, BMI, and academic performance. Our findings contribute to the growing body of research on executive function and its long-term effects on behavior and health outcomes.

### 4.1 Factor structure of executive function and impulsivity

The four-factor solution showed the best fit indices of all models and was chosen based on the scree plot and parallel analysis, providing the most parsimonious and theoretically meaningful model comprised of factors reflecting adaptive and emotional impulsivity, verbal comprehension/language, and attention/memory/executive function.

The first factor, comprising the UPPS-P Lack of Perseverance and Lack of Planning subscales, aligns with previous research(Friedman and Robbins, 2022) suggesting that difficulty in maintaining goal-directed behavior and planning are core components of impulsivity and executive dysfunction. The factor structure highlights the role of impulsivity in impairing higher-order cognitive processes such as planning and perseverance, which are crucial for adaptive behavior.

The second factor, comprising the UPPS-P Positive Urgency, Negative Urgency, and Sensation Seeking subscales, captures impulsivity related to emotional reactivity. This second factor indicates that individuals who exhibit high levels of urgency are more prone to making impulsive decisions under emotional distress, consistent with theoretical models of affect-driven impulsivity(Hinojosa-Aguayo and González, 2020; Muela et al., 2023). These findings align with studies showing that emotional impulsivity is associated with maladaptive behaviors such as substance use and binge eating(Derks et al., 2022; Howells et al., 2024).

The third factor, comprising cognitive measures from the NIH Toolbox, reflects Verbal Ability and Cognitive Control. Specifically, Picture Vocabulary, List Sorting, and Reading, highlighting the importance of cognitive flexibility, inhibitory control, and verbal comprehension as core aspects of executive function. This factor supports the notion that verbal ability and cognitive control are interconnected, facilitating goal-directed behavior and effective decision-making(Jin et al., 2019; Toplak et al., 2010).

The fourth factor includes measures related to Flanker Inhibitory Control, Card Sorting and Pattern Comparison, which appear to tap into working memory and visual processing. These cognitive abilities are fundamental for information retention and manipulation, necessary for tasks requiring organization and pattern recognition(Cowan, 2014; Gathercole et al., 2019; Jablonska et al., 2020). Taken together, these four factors explain a significant portion of the variance in cognitive function and impulsivity, reinforcing the multidimensional nature of these constructs.

### 4.2 Factor mediation and moderation of physical activity, BMI, and academic performance

The structural equation model (SEM) demonstrated strong overall fit and the three latent factors—Adaptive Impulsivity, Emotional Impulsivity, and Memory/Attention/Executive Function—were tested as predictors of multiple outcomes, including physical activity, body mass index (BMI), and academic performance.

In addition to the direct paths between impulsivity traits and outcomes, the model explored Peer Network Health and Perceived Stress as potential mediators and Parental Monitoring as a potential moderator of these relationships.

#### 4.2.1 Peer network health as a mediator

Peer Network Health played a significant mediating role between impulsivity traits and physical activity. Peer Network Health was positively influenced by Factor 2 (Emotional Impulsivity) (β = 1.046, p = 0.001), indicating that individuals with stronger emotional impulsivity traits may cultivate more protective peer networks. This finding suggests that peers may provide a social buffering effect, helping individuals manage impulsive tendencies or emotional challenges. Importantly, Peer Network Health also positively influenced Physical Activity (β = 0.004, p = 0.015), suggesting that strong peer relationships encourage greater engagement in physical activities. Thus, Peer Network Health functioned as an important mediator in the pathway between emotional impulsivity and physical activity, emphasizing the role of social connections in promoting healthier behaviors.

However, the relationship between Factor 1 (Adaptive Impulsivity) and Peer Network Health was negative and significant (β = −0.812, p = 0.009), suggesting that individuals with traits linked to task persistence or planning difficulties may have less protective peer networks. This highlights a potential distinction between emotional and adaptive impulsivity, where emotional impulsivity fosters stronger social networks, but difficulties in perseverance and planning may strain peer relationships.

#### 4.2.2 Perceived stress as a mediator

Perceived Stress did not significantly mediate the effects of impulsivity traits on academic performance and BMI as initially expected. Factor 1 (Adaptive Impulsivity) was not significantly associated with Perceived Stress (β = 1.122, p = 0.568), and Perceived Stress did not show a significant influence on Past Year Grades (p = 0.080) or BMI (p = 0.916). These results suggest that, contrary to the initial hypothesis, emotional dysregulation, as captured by impulsivity traits, did not increase perceived stress levels in a way that meaningfully impacted academic performance or health-related behaviors. However, physical activity and academic performance were more strongly influenced by Peer Network Health, indicating that social support might be more critical than stress in this context.

#### 4.2.3 Parental monitoring as a moderator

Parental Monitoring was incorporated into the model as a moderator, and its protective role was particularly evident for Factor 2 (Emotional Impulsivity). It moderated the effects of emotional impulsivity on both physical activity and grades, weakening the negative impact of emotional impulsivity on these outcomes. For instance, with stronger Parental Monitoring, the negative relationship between emotional impulsivity and academic performance (β = 0.267, p < 0.001) was mitigated, reducing the potential for impulsive behaviors to harm academic outcomes. This underscores the importance of parental involvement in buffering some of the negative effects of emotional dysregulation, especially in academic contexts.

The effects of Factor 1 (Adaptive Impulsivity) were more complex. While the direct effect of Factor 1 on study outcomes was negative, particularly for physical activity (β = −0.036, p = 0.010), Parental Monitoring also provided a protective moderating effect. For example, interactions involving parental monitoring (β = 0.033, p = 0.006) helped counterbalance some of the adverse effects of Factor 1 on physical activity. This suggests that, even when impulsivity traits related to lack of perseverance and planning are present, increased parental oversight can help mitigate their negative influence on both health and academic behaviors. Thus, Parental Monitoring remains a key intervention point for reducing the harmful effects of impulsive tendencies, especially in educational and physical health settings.

#### 4.2.4 Direct effects and implications

The model revealed a significant positive relationship between emotional impulsivity and academic performance (β = 0.267, p < 0.001), suggesting that higher emotional impulsivity was associated with better academic performance (since the grade scale is inversely coded, lower values reflect higher grades). This finding suggests that, in certain contexts, emotional impulsivity may be linked to adaptive strategies that enhance academic success, such as quick responsiveness to feedback or risk-taking behaviors that promote engagement and learning.

In contrast, Factor 1 (Adaptive Impulsivity) showed a weaker, negative relationship with academic performance (β = −0.046, p < 0.001), indicating that higher levels of this form of impulsivity were associated with poorer academic outcomes. This aligns with prior research suggesting that while adaptive impulsivity may support task persistence(Fortgang and Cannon, 2022; Lunkenheimer et al., 2019), it can interfere with structured academic settings, where impulsive decision-making(Hinojosa-Aguayo and González, 2020) may detract from careful, methodical learning(Abdulghani et al., 2014).

Furthermore, the relationships between impulsivity traits and physical activity were significant. Emotional Impulsivity positively predicted higher physical activity levels (β = −0.036, p = 0.010), while the interaction between parental monitoring and impulsivity also influenced activity levels. This suggests that individuals with emotional impulsivity may be more inclined to engage in physical activities(Cheval et al., 2016; Ghahramani et al., 2016), possibly due to a preference for stimulation or dynamic environments.

These findings support the idea that impulsive individuals, especially those high in emotional impulsivity, may gravitate toward high-energy activities(Smith et al., 2021) and perform better academically in certain contexts, particularly when external support systems like parental monitoring(Hill and Tyson, 2009; Lopez-Tamayo et al., 2016) are in place.

#### 4.2.5 BMI and health-related behaviors

The model also identified significant associations between impulsivity traits and BMI. Emotional Impulsivity was positively associated with higher BMI (β = 0.097, p < 0.001), suggesting that difficulties with emotional regulation may contribute to behaviors that promote weight gain, such as impulsive eating or poor dietary control. However, the model did not find a significant mediation effect of Perceived Stress on BMI (p = 0.916), indicating that stress did not play a prominent role in linking impulsivity to weight gain in this context. Instead, direct associations between impulsivity traits and BMI highlight how emotional dysregulation may lead to less healthy behavioral patterns, contributing to elevated BMI.

Overall, these findings emphasize the multifaceted role of impulsivity in shaping adolescents' physical, social, and academic outcomes. Notably, Peer Network Health emerged as a critical protective factor, helping mitigate the adverse effects of impulsive traits on behaviors such as physical activity. This underscores the importance of fostering supportive social relationships as a key intervention strategy for managing impulsivity and its potential consequences. These insights have significant implications for interventions aimed at improving adolescent health and academic performance, suggesting that building strong peer networks may be crucial in helping adolescents cope with impulsivity-related challenges.

Despite the strengths of this study, several limitations must be acknowledged. First, the cross-sectional nature of the EFA/CFA limits our ability to draw causal inferences, though the longitudinal nature of the SEM analyses using ABCD data from Years 1 through 5 partially addresses this weakness. It is also possible that unmeasured confounding variables influenced the observed associations. Second, the reliance on self-report measures (e.g., UPPS subscales, Parental Monitoring) may introduce bias, as participants may under- or over-report their behaviors. We were not able to include Factor 3 in the SEM due to lack of convergence despite considerable efforts of fixing variances as well as removal of small to negative variances between factors. While the four-factor model explained a substantial proportion of the overall variance, there may be additional factors contributing to executive function that were not captured by the measures used in this study. The scores across the two methods of objective cognitive testing (i.e., NIH toolbox cognition battery) and self-report (i.e., impulsivity) do not co-vary, and this could be due to method effects or trait effects(Campbell and Fiske, 1959). However, the results are helpful and informative for those looking to create composites using NIH toolbox cognitive and impulsivity assessments. Lastly, but perhaps most notably, because of the low prevalence of other health behaviors such as alcohol and other substance use, we were not able to include these as outcomes in the constructed SEM.

The current study highlights the intricate relationships between different domains of impulsivity, cognitive functioning, and key life outcomes such as academic performance, physical activity, and BMI. Peer Network Health emerged as a significant mediator, particularly between Emotional Impulsivity and physical activity, emphasizing the importance of social contexts in shaping how impulsivity affects both academic and health outcomes. Contrary to initial expectations, Perceived Stress did not play a major mediating role in the pathways linking impulsivity to BMI or academic performance.

The finding that emotional impulsivity was positively associated with academic performance (despite the inverse grade scale) suggests a potential adaptive aspect of emotional impulsivity in certain contexts, such as responsiveness to feedback or engagement in dynamic environments. In contrast, Factor 1 (Adaptive Impulsivity) was associated with poorer academic outcomes, reflecting the complex and sometimes contradictory effects of impulsivity traits depending on the specific outcome.

Parental Monitoring played a moderating role, buffering the negative impacts of impulsivity on both academic performance and physical activity. This finding underscores the potential for family-based interventions to help mitigate the negative consequences of impulsive behavior, particularly by fostering more structured environments that can help adolescents manage impulsive tendencies.

The study also demonstrated that emotional impulsivity is linked to higher BMI, suggesting that difficulties with emotional regulation may contribute to unhealthy behavioral patterns such as impulsive eating. However, Perceived Stress did not emerge as a significant mediator in this relationship, pointing instead to the direct effects of impulsivity traits on health-related outcomes like BMI.

In conclusion, this study provides an important contribution to understanding the multidimensional effects of impulsivity and the mediating role of Peer Network Health, with Parental Monitoring acting as a key moderator. While further research is needed to address the limitations and expand on these findings, the results suggest that interventions aimed at improving emotional regulation, strengthening peer relationships, and enhancing parental involvement could lead to better academic and health-related outcomes in individuals with high impulsivity traits.

## CRediT authorship contribution statement

**Vijay A. Ramchandani:** Funding acquisition, Supervision, Writing – review & editing. **Michael L. Thomas:** Formal analysis, Methodology, Supervision, Writing – review & editing. **Tommy Gunawan:** Formal analysis, Methodology, Visualization, Writing – review & editing. **Alejandro Daniel Meruelo:** Conceptualization, Formal analysis, Funding acquisition, Writing – original draft, Writing – review & editing.

## Declaration of Competing Interest

The authors declare that they have no known competing financial interests or personal relationships that could have appeared to influence the work reported in this paper.

## Data Availability

Available via NDA and described in data statement.

## Appendix A Supplementary material

Supplementary material

Supplemental Figure 1: Study Timeline Assessment Data Collection by Year A schematic of study timeline assessment data collection by year for both cognitive and impulsivity measures, and additional data on variables for the mediation-moderation structural equation model analyses
