# Supplementary material for: A four-factor model of executive function: Predicting physical and academic outcomes from cognitive assessments in adolescents
Source: Dev Cogn Neurosci. 2024 Oct 30;70:101471. doi: 10.1016/j.dcn.2024.101471 (PMC11566708; doi:10.1016/j.dcn.2024.101471)
Supplement: Supplementary file 1 — Supplementary material [file mmc1.docx]

**Supplemental Table 1:** Factor Loadings for the 2-Factor Solution

| **Measure** | **Factor 1** | **Factor 2** |
| --- | --- | --- |
| UPPS Lack of Perseverance | -0.14 | 0.43 |
| UPPS Positive Urgency | -0.11 | 0.67 |
| UPPS Sensation Seeking | 0.15 | 0.26 |
| UPPS Negative Urgency | -0.03 | 0.65 |
| UPPS Lack of Planning | 0 | 0.52 |
| NIH Tbx Picture Vocabulary (Age Corrected) | 0.53 | -0.06 |
| NIH Tbx Flanker (Age Corrected) | 0.49 | 0.04 |
| NIH Tbx List (Age Corrected) | 0.55 | -0.08 |
| NIH Tbx Card Sort (Age Corrected) | 0.59 | 0 |
| NIH Tbx Pattern (Age Corrected) | 0.45 | 0 |
| NIH Tbx Picture (Age Corrected) | 0.41 | -0.08 |
| NIH Tbx Reading (Age Corrected) | 0.52 | -0.04 |
| Delayed Discounting K-value | 0.01 | 0 |

*NIH Tbx - NIH Toolbox*

The two-factor solution demonstrated an adequate fit, with 53 degrees of freedom for the model and an objective function value of 0.54. The root mean square of the residuals (RMSR) was 0.06, and the df-corrected RMSR was 0.08, indicating a reasonable fit. The RMSEA index was 0.098, with a 90% confidence interval ranging from 0.094 to 0.103, suggesting modest model fit. The Tucker-Lewis Index (TLI) of factoring reliability was 0.623, and the Bayesian Information Criterion (BIC) was 787.23. Additionally, the fit based upon off-diagonal values was 0.88.

Factor 1 captured variance in cognitive measures from the NIH Toolbox Cognition Battery, such as Picture Vocabulary, Flanker, List Sorting, and Card Sort, with loadings ranging from 0.41 to 0.59. Factor 2 was associated with impulsivity-related measures from the UPPS-P Impulsive Behavior Scale, including Lack of Perseverance, Positive Urgency, Negative Urgency, and Lack of Planning, with loadings of 0.43 to 0.67. These two factors accounted for 25% of the total variance, with Factor 1 explaining 14% and Factor 2 contributing 11%. These findings suggest a meaningful distinction between the cognitive and impulsivity-related domains captured by the two factors.

**Supplemental Table 2:** Factor Loadings for the 3-Factor Solution

| **Measure** | **Factor 1** | **Factor 2** | **Factor 3** |
| --- | --- | --- | --- |
| UPPS Lack of Perseverance | -0.12 | 0.43 | -0.07 |
| UPPS Positive Urgency | -0.13 | 0.67 | -0.02 |
| UPPS Sensation Seeking | 0.15 | 0.27 | 0.04 |
| UPPS Negative Urgency | -0.04 | 0.66 | -0.01 |
| UPPS Lack of Planning | -0.01 | 0.52 | 0 |
| NIH Tbx Picture Vocabulary (Age Corrected) | 0.73 | -0.02 | 0.03 |
| NIH Tbx Flanker (Age Corrected) | 0.19 | 0.02 | 0.57 |
| NIH Tbx List (Age Corrected) | 0.53 | -0.06 | 0.2 |
| NIH Tbx Card Sort (Age Corrected) | 0.26 | -0.02 | 0.67 |
| NIH Tbx Pattern (Age Corrected) | 0.15 | -0.03 | 0.56 |
| NIH Tbx Picture (Age Corrected) | 0.33 | -0.08 | 0.22 |
| NIH Tbx Reading (Age Corrected) | 0.63 | -0.01 | 0.08 |
| Delayed Discounting K-value | 0 | 0 | 0.03 |

*NIH Tbx - NIH Toolbox*

The three-factor solution demonstrated adequate fit, with 42 degrees of freedom for the model and an objective function value of 0.27. The root mean square of the residuals (RMSR) was 0.04, and the df-corrected RMSR was 0.06, indicating a good fit. The RMSEA index was 0.077, with a 90% confidence interval ranging from 0.071 to 0.082, suggesting a modest model fit. The Tucker-Lewis Index (TLI) of factoring reliability was 0.771, and the Bayesian Information Criterion (BIC) was 268.8. Additionally, the fit based upon off-diagonal values was 0.94.

Factor 1 primarily captured variance in cognitive measures from the NIH Toolbox Cognition Battery, such as Picture Vocabulary, Reading, and List Sorting, with loadings ranging from 0.53 to 0.73. Factor 2 was associated with impulsivity-related measures from the UPPS-P Impulsive Behavior Scale, including Lack of Perseverance, Positive Urgency, and Lack of Planning, with loadings ranging from 0.43 to 0.67. Factor 3 captured variance in memory/attention/executive function measures such as Flanker and Card Sort, with loadings of 0.57 and 0.67, respectively. These three factors accounted for 31% of the total variance in the data, with Factor 1 explaining 11%, Factor 2 contributing another 11%, and Factor 3 accounting for 9%. These findings suggest a meaningful distinction between the cognitive, impulsivity-related, and executive function domains captured by the three factors.
