# Supplementary figures and images for: A four-factor model of executive function: Predicting physical and academic outcomes from cognitive assessments in adolescents
Source: Dev Cogn Neurosci. 2024 Oct 30;70:101471. doi: 10.1016/j.dcn.2024.101471 (PMC11566708; doi:10.1016/j.dcn.2024.101471)

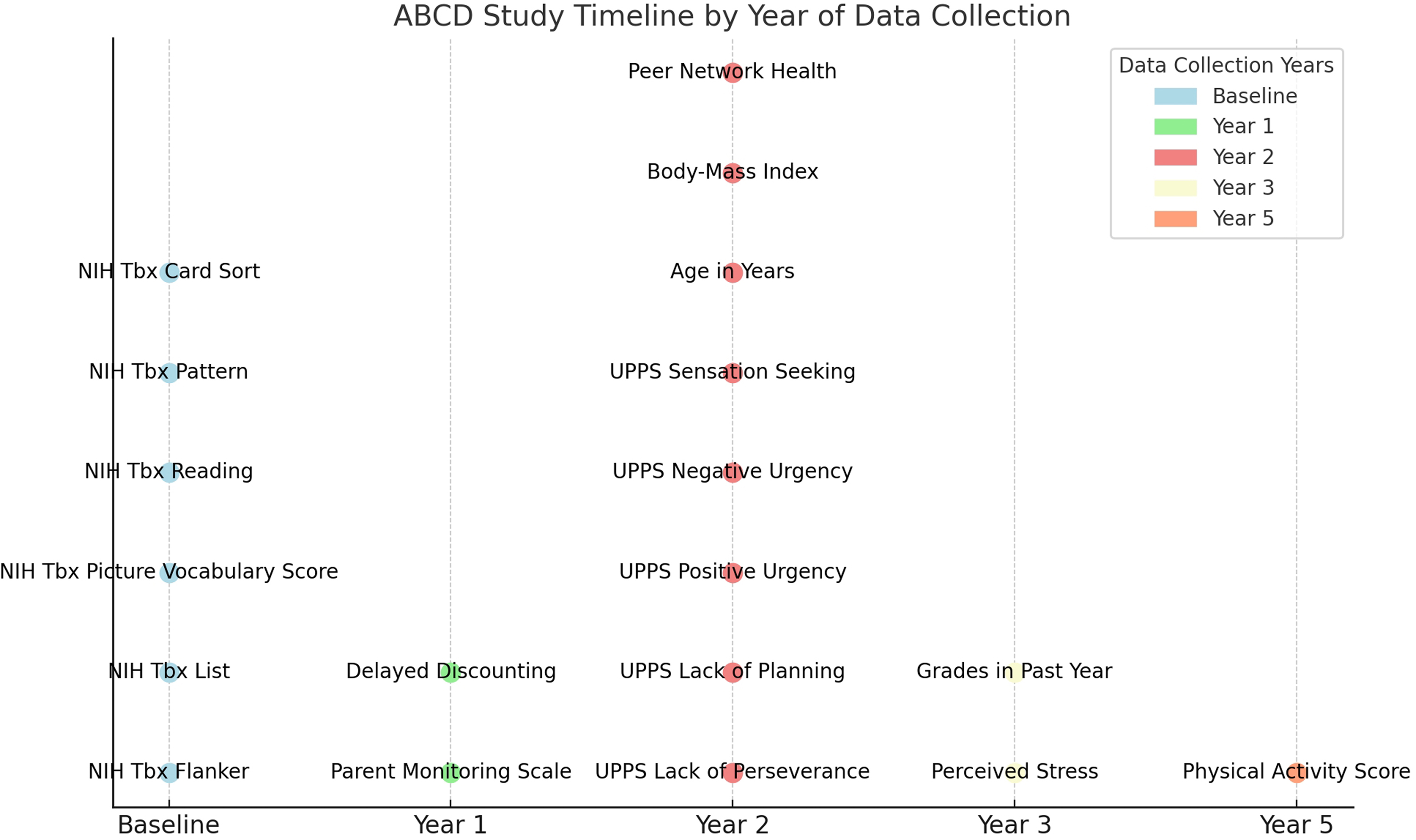

Supplement: Supplementary file 2 — Supplemental Figure 1: Study Timeline Assessment Data Collection by Year A schematic of study timeline assessment data collection by year for both cognitive and impulsivity measures, and additional data on variables for the mediation-moderation structural equation model analyses [file mmc2.jpg]
